# Multifunctionality of Thermal Protective Layer Interchanging Double Cloth Conditioned by Influential Parameters

**DOI:** 10.3390/polym14214561

**Published:** 2022-10-27

**Authors:** Tea Badrov, Ivana Schwarz, Stana Kovačević

**Affiliations:** Department of Textile Design and Management, Faculty of Textile Technology, University of Zagreb, Prilaz Baruna Filipovića 28a, 10000 Zagreb, Croatia

**Keywords:** aramid and modacrylic/cotton fibres, yarn fineness, multifunctional protective woven fabric, thermal and thermo-physiological properties

## Abstract

The proportion of woven fabrics in the broad field of protective textiles is extremely high. By various procedures (surface treatments, fabric lamination, composite production), fabric properties that meet the requirements defined by standards are achieved. However, simultaneously, these procedures cause negative effects in the form of fabric thickness, stiffness, impermeability, non-breathability, and thus, discomfort. Therefore, there are valid and justified reasons to approach the design process of making such woven fabrics using more complex construction solutions—layer interchanging double cloth. In addition, by applying fibres with integrated desired properties and other structural fabric parameters, it is possible to influence the achievement of the properties of multi-purpose multilayer fabrics for protection, which is the aim of this research. The application and combination of aramid and modacrylic/cotton fibres and use of different yarn fineness resulted in different intensities of protection. The correlative values of mentioned parameters and thermal and thermo-physiological properties indicate their strong connection, and thus the effectiveness of the developed woven fabric samples.

## 1. Introduction

The world of traditional textiles has changed throughout history because of cultural influences and technological innovations. Research technologies have led to the development of woven fabrics to the point where they can meet the functional goals of products beyond the traditional role of clothing or household items. The original properties of textile use and production, body protection (from external influences), developed due to technological achievements and application needs in extreme situations, but also necessary aspects of protection (protective clothing).

Fabrics used for protective clothing represent one of the most specific and market-significant niches in the global market of textile products, with a large number of woven fabrics intended for protection from various influences. This area also includes clothing, i.e., woven fabrics with the fundamental role of protecting the body from burning during various activities [1].

The achievement of key properties of woven fabric, as the most complex textile structure, is influenced by properties at a macro-, meso-, and micro-level. Macro, as one of the most important levels of woven fabric that affects its overall properties, is a construction specification, including parameters such as weave, thickness, mass, and fabric density. Fabric weave forms the properties of thickness and mass, but on the other hand, it determines water-vapour permeability and thermal insulation properties. The meso-level includes the yarn’s structural parameters. This primarily refers to yarn fineness, which, in combination with other structural yarn parameters, affects its mechanical properties [2]. Micro-level implies the fibre properties, which directly affect the meso-level, and thus the macro-level, of the woven fabric. This makes it clear that fibres carry the fundamental properties of any woven fabric.

Thermal protective woven fabric must perform two opposing functions—it must prevent external heat from flowing towards the body, and at the same time allow metabolic heat to escape to the atmosphere, to avoid heat stress and stress-related heart attack. Current firefighters’ protective clothing protects them from external heat and moisture while simultaneously preventing the wearer’s heat and moisture to flow from the body. The trapped amount of heat and moisture, in the wearer’s protective clothing causes the body temperature to rise rapidly, directly affecting the risk of steam burn injuries and heat stress. Heat stress is a common problem for firefighters. When exposed, their body activates sweat glands and starts to produce sweat. The sweat accumulates on the skin’s surface and gradually dissipates into the surrounding environment. By converting sweat into vapour, the dissipation process cools down the body by absorbing its metabolic heat. The bigger the amount of heat and moisture that is trapped in the protective clothing, the larger the risk of injury. This is why it is crucial that protective clothing simultaneously protects the wearer from external heat and releases the body’s excess metabolic heat into the environment [3,4,5].

On the other hand, these conditions frequently result in an imbalance between body heat generation and heat loss, increasing core body temperature (hyperthermia). Heat balance is influenced by a variety of factors that can be divided into three categories: environmental (humidity, air temperature, radiation temperature, etc.), physiological (metabolic heat), and clothing (clothing insulation). Since the definition of thermal comfort is “a subjective expression of satisfaction with the thermal environment”, the required conditions for thermal comfort are not the same for everyone. Having this in mind, the wearer’s thermal comfort and performance have an important role in protective clothing in high-risk professions such as firefighting [6,7]. Therefore, when designing such clothing, there should be a balance between high thermal comfort and high thermal protection, including sufficient thermal transfer and water-vapour permeability. These conflicting and challenging requirements explain the continuous development of protective clothing that would optimize the balance between comfort and protection [8,9,10]. Certain parameters such as fibre morphology, structural and physical material properties, and finishing influence the amount of thermal protection of textile materials. Each parameter is affected by the fibre type, yarn properties (i.e., the spinning technique, yarn fineness, etc.), fabric composition, and the finishing process. One cause of firefighter injuries and deaths is an improper evaluation of the thermal protection performance of fabric systems. This is why, when it comes to the development of efficient fire-protective textile materials, one of the most important steps is to select the proper raw material [11,12,13].

The importance of woven fabrics for thermal protection imposes the need for the invention and application of an economical and ecologically favourable way of developing materials with improved FR properties and reduced flammability. Protective woven fabrics of reduced flammability, or non-flammable fabrics (FR, Flame Retardant) for targeted purposes, are mostly composite and laminate materials, which represent a compact flat composition composed of several diverse materials. Surface treatment of single-layer fabrics, i.e., their impregnation, is necessary to achieve complete protection from external influences. The most durable treatment is achieved by using organophosphorus compounds. However, this process has a major disadvantage of FR property loss with washing. The best non-flammability properties are achieved by using synthetic materials obtained by adding special additives to the melt or solution before spinning, which improve the FR properties [14,15].

Firefighter clothing is a multilayer garment which normally consists of three layers, i.e., outer shell, moisture barrier, and thermal barrier [16]. By layering woven fabrics, it is possible to fulfil the requirements set by standards, but at the same time, the material is thick, which causes an increase in impermeability, resulting in extreme discomfort when wearing, i.e., a decrease in comfort. The property of insufficient comfort of the material is a consequence of the property of non-breathability. Multi-layer protective clothing contains lower layers that are joined to the upper woven fabric layer by a sewing process. Their structure, raw material, and properties differ from woven fabrics, which results in different behaviour in real application conditions. Composite materials used for protective purposes represent a compact flat structure composed of several different materials. Mostly, these are sandwich forms, where the woven fabric forms the top layer, as a basic material that has satisfactory properties, while the other layers, different flat structures, have noticeably weaker physical and mechanical properties. Due to their stiffness, mass, and thickness, composite materials are not acceptable for the production of the entire protective clothes, but are used for certain parts that are most exposed to danger [17,18,19,20,21,22].

Today, it is increasingly demanding, complex, and difficult to achieve certain properties of woven fabric or composites defined by standards, and it is even more difficult to achieve the sustainability of these properties during use. In addition to the ability to provide a high level of protection, woven fabrics for protective clothing must be characterized by comfort and easy maintenance and cleaning. The basic requirements for protective clothing are harmlessness (it must not adversely affect health, which represents a major challenge due to the use of numerous chemical substances on raw materials and surface treatments), design (with the use of appropriate woven fabric, to ensure perfect movement of the body during wearing and performing work tasks), and comfort (feeling of ease and comfort that affects the psycho-physical state) [23].

For the fabric intended to protect the body to meet the overall properties defined by the standards, special attention is paid to functionality and efficiency. For this purpose, research will be carried out on the design of complex woven fabrics (layer interchanging double cloth), using fibres of different raw materials compositions and with integrated protection properties. Such fabric structure reflects diversity on the front and backside of the fabric, achieving the properties of multi-functionality by meeting the mechanical, thermal and thermo-physiological requirements, all in one fabric, produced in a single process (cost-efficiency and environmentally friendly).

This research will expand knowledge in the field of so-called layer interchanging double cloth, which is poorly represented in application and science compared to conventional single-layer woven fabrics. The reason for this is the extreme complexity of the structure and the comprehensive knowledge necessary for its development, production. and application.

Layer interchanging double cloth is created by the simultaneous production of two woven fabrics on one loom, which are interlaced with warp or weft threads from the upper fabric to the lower one or vice versa. The interlacing threads can differ in raw material, fineness and production technology compared to other threads, or they can be the same as the threads of the base fabrics. With such fabrics, it is important to coordinate the weave of the upper and lower fabric so that the interlacing threads are not visible on the front, nor on the backside. This weaving technique is one of the most complex, and the development of such fabrics requires exceptional professional and technological knowledge. To achieve the desired properties, it is necessary to carry out studious analyses when designing such fabrics. The goal is to meet the key properties of non-flammability and protection on the fabric front side and comfort on the fabric backside. Due to their multi-functional properties, such woven fabrics deserve much greater attention and application in many areas of technical fabrics, especially for protective clothing.

Each layer in the current multi-layer protective clothing has to be separately produced and then assembled together. Compared to current multi-layer protective clothing, layer interchanging double cloth is more cost-efficient and eco-friendly because of the less-demanding production process—one machine simultaneously produces two interlaced woven fabrics. Also, costs are lower when it comes to prototype testing. Using fewer resources, it provides a base to develop more effective thermal protective clothing at a lower cost.

Therefore, the aim of this research is to investigate the multifunctional properties of thermal protective layer interchanging double cloth conditioned by influential parameters, by using a complex woven structure, certain raw materials, and varying yarn fineness.

## 2. Materials and Methods

To achieve certain woven fabric protection properties, the fibres used for production were carefully selected. One of those fibres is 95% poly (meta-phenylene isophthalamide) (m-aramid) blended with 5% poly (para-phenylene isophthalamide) (p-aramid), both of which have an important role in the segment of protective clothing. These fibres are best known for their combination of fire resistance, great thermal properties (stability, conductivity, and resistance), and excellent mechanical properties. In addition, meta-aramid fibres do not ignite, melt, or drip, a major reason for their success in the fireproof apparel market. In comparison to commodity fibres, m-aramids provide better long-term retention of mechanical properties at elevated temperatures. When combined with other fibres such as para-aramid, the breaking strength increases.

Furthermore, a mixture of FR modacrylic fibres and cotton fibres was used to achieve another aspect of the protective woven fabric’s functionality, which is comfort, lightness, and softness. Modacrylics, unlike other inherent FR fibres, can be blended with natural fibres. Modacrylic fibres are able to remove oxygen from non-FR fibres. This means they can protect themselves, as well as the other fibres in the blend, from overheating and burning. Due to the comfort qualities of cotton, the mix with cotton results in a more comfortable fabric. Table 1 shows the declared parameters of the used yarns.

The fabric weave, in addition to yarn fineness, affects the properties of fabric thickness and mass, but on the other hand, it determines the vapour permeability and thermal insulation properties. This is why woven fabric samples used in this research are so-called layer interchanging double cloth. Such woven fabrics are characterized by an extremely complex structure.

For the purpose of this research, 16 fabrics were woven, using the same weave and yarns of the previously mentioned types of fibres, keeping the warp threads’ fineness constant, but combining the fineness of the weft threads. The weaving process was conducted on the laboratory weaving machine FYI DW598. The specification of the woven fabric samples is shown in Table 2.

A complete evaluation of the basic construction and structural parameters of the woven fabric samples was performed using standardized methods. These include fabric mass per unit area (ISO 3801:1977), fabric thickness (ISO 5084:1997) (under a load of 1 kPa) and fabric density (ISO 7211-5:2020). The properties that contribute to the physiological comfort of textile materials involve a certain combination of heat and mass transfer. Thermo-physiological properties—thermal properties and water-vapour resistance of the samples—were determined using the standardized method ISO 11092:1993 (sweating guarded-hotplate test) on the instrument MTNW-Thermetrics, SAD, SGHP-8.2. The thermal protective effect (resistance to radiant heat) of woven fabric samples was determined using the standardized method ISO 6942:2002, Method B, performed on TE-08 Radiant Heat Exposure Tester. Radiation heat transfer was tested using a default heat flux of Q_0_ = 20 kW/m^2^. Samples were conditioned before testing for at least 24 h at a temperature of 20 ± 2 °C and relative humidity of 65 ± 5%, tested on the fabric front-side. All tests were carried out in an accredited laboratory.

## 3. Results and Discussion

**Figure 1:**
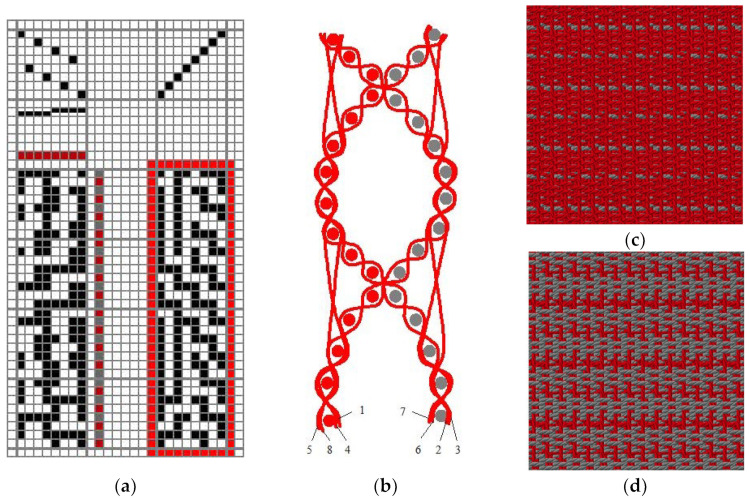
Structure of the layer interchanging double cloth: (**a**) fabric weave, (**b**) fabric cross-section, (**c**) fabric front side, (**d**) fabric backside.

Samples produced for this research represent a complex woven structure, so-called layer interchanging double cloth. All fabrics are composed of the same thread used for the warp system: aramid yarn (AR), a fineness of 17 × 2 tex. For the weft thread system, two yarns of different raw materials were used: aramid yarn (AR) and modacrylic/cotton yarn (MC) with different finenesses (20 × 2, 17 × 2, 14 × 2, 12.5 × 2 tex). Due to the specific fabric construction, i.e., the weave (Figure 1a), with the specific programming of the weft selection, the predominance of aramid yarn on the front side (Figure 1c) and modacrylic/cotton yarn on the backside (Fabric 1d) of the fabric is achieved (where red colour represents aramid yarn and grey colour modacrylic yarn). The predominance of aramid yarn on the front side (top surface) was chosen, like in the outer shell of multilayer clothing, because of its fire resistance, great thermal properties and excellent mechanical properties. As for the backside (bottom surface), the predominance of modacrylic/cotton yarn was used to achieve functionality (comfort, lightness, and softness). Figure 1b shows the fabric cross-section, with numerations 1, 4, 5, and 8 as warp threads of the front side fabric, and 2, 3, 6, and 7 as warp threads of the backside fabric. Threads 4 and 8 on the fabric front side, and threads 2 and 6 on the fabric backside show the interlacing threads. Yarn properties conditioned by the raw material composition and inherent properties affect the properties of the entire fabric, thus achieving multi-functionality by meeting the mechanical, thermal, and thermo-physiological requirements defined for this type of fabric.

Table 3 shows that the warp density stays constant for all samples (34 threads/cm), while the weft densities vary from 58–69 threads/cm. In accordance with the change in yarn fineness, the masses of the samples change as well as the fabric thickness. By increasing the fineness of an individual component, the fabric mass and thickness decrease.

The results of the resistance to the radiant heat of the woven samples are shown in Table 4. The transmitted heat flux density (Q_c_, kW/m^2^) was calculated according to the equation:

(1) $$Q_{c}=\frac{M\cdot C_{p}\cdot 12}{A\;\cdot \;\left(t_{24}-t_{12}\right)}$$

A high heat transmission factor (TF(Q_0_)) indicates good heat transfer, while a low heat transmission factor indicates good insulation. The factor of heat transfer was calculated according to the equation [24]:

(2) $$\mathrm{TF}\left(Q_{0}\right)=\frac{Q_{c}}{Q_{0}}$$

It is important to emphasize that the results of the tested parameters of resistance to the radiant heat of all tested samples (shown in Table 4) meet the requirements according to the standard EN ISO 15384:2020 (Protective clothing for firefighters—Laboratory test methods and performance requirements for wildland firefighting clothing), which defines that t_24_ ≥ 11 s, t_24_ − t_12_ ≥ 4 s, and TFQ_0_ ≤ 0.700.

**Figure 2:**
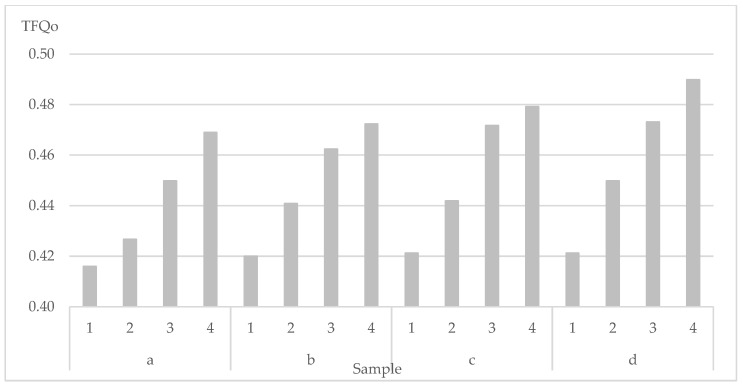
Heat transmission factor influenced by weft yarn fineness and raw material.

Furthermore, the impact of yarn fineness and raw material in the weft thread system on the heat transmission factor is evident. By grouping the samples according to the combination of fibre type and fineness (Figure 2), where MC yarn fineness is constant while the fineness of AR yarn is diverse, there is a clear difference in the heat transmission factor value.

Within a certain group, the influence caused by the change in the AR yarn fineness is visible: due to its increase, the heat transmission factor also increases. This increase within the groups ranges from 11.1% to 14.0%.

Furthermore, between the groups, an additional influence due to the change in the MC yarn fineness is also visible, where as a result of its increase, the heat transmission factor also increases. This increase is lower and ranges from 1.3% to 5.2%.

The total difference in the heat transmission factor between samples 1a and 4d amounts to 15.1% in total.

The above-mentioned results prove that a change of AR yarn fineness has a greater influence on heat transmission factor, and thus the thermal insulation properties. The increase of its fineness also increases heat transmission factor, while decreasing thermal insulation properties of a woven fabric.

**Figure 3:**
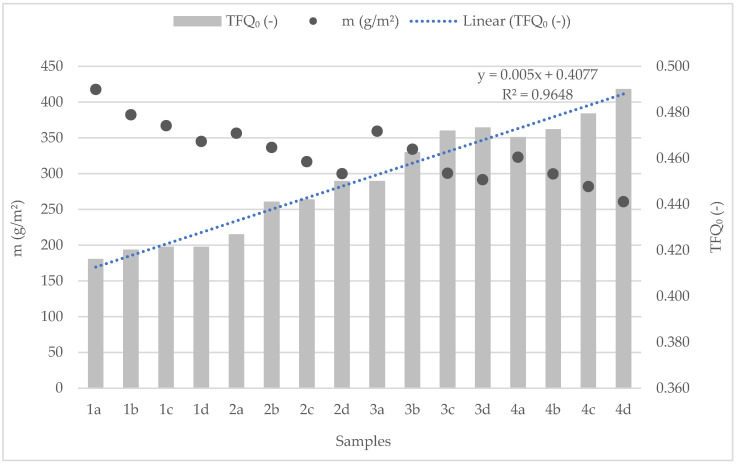
Heat transmission factor compared with woven fabric mass.

Figure 3 shows the results of the heat transmission factor (TFQ_0_) of the tested fabric samples in comparison to mass. By increasing the weft thread fineness, i.e., reducing fabric mass, the heat transmission factor changes inversely proportionally. Sample 1a, with the highest mass (the lowest fineness in the weft system (AR 20 × 2 tex + MC 20 × 2 tex)), results in a heat transmission factor of 0.42, while Sample 4d, with the lowest mass (highest thread finesses used in weft system (AR 12.5 × 2 tex + MC 12.5 × 2 tex)), results in a heat transmission factor of 0.490. This shows that samples with lower yarn fineness (i.e., highest mass) provide better insulation properties, while the ones with higher fineness show better heat transfer through the sample to the calorimeter. By interrelating the properties of the tested sample’s heat transmission factor (TFQ_0_) with fabric masses, the high correlation between the above is clearly stated (R^2^ = 0.9648).

The results of the tested thermal resistance (R_ct_) property, which according to the standard EN ISO 15384:2020 (Protective clothing for firefighters—Laboratory test methods and performance requirements for wildland firefighting clothing) should satisfy the requirement of R_ct_ ≤ 0.055, point to the limit values, with minor deviations (Table 5).

The results mainly depend on the yarn fineness, so that the finer yarn, which often has a higher density, provides greater thermal resistance. Since the coarser weft yarn (20 × 2 tex) affected the greater fabric mass and thickness on some samples, the thermal resistance turned out to be higher (samples 1a and 3a). Samples from groups 3 and 4 with the finest weft yarns provide almost the lowest thermal resistance, which points to the fact that finer and denser yarns in weft systems are not always a guarantee of greater thermal protection, and that these samples with reduced mass and thickness provide less thermal resistance. It can be concluded that a finer and denser yarn in the weft system allows better protection to some extent when the reduced mass and thickness begin to disrupt that continuity. To understand the real differences in the property of thermal resistance, the role of the pores in the woven fabric, which can greatly influence this property with their number, shape, and size, is of great importance. Observing the samples from group 4, samples 4b (0.0632) and 4c (0.0631) show the highest thermal resistance, which points to the fact that when designing the fabric, it is necessary to optimize the results by taking into account the structural parameters (yarn fineness, fabric density, mass, and thickness) and, finally, choose samples with the highest efficiency.

The results of water-vapour resistance properties (R_et_) show that all tested samples meet the requirements set by EN ISO 15384:2020 standard (Protective clothing for firefighters—Laboratory test methods and performance requirements for wildland firefighting clothing), which defines R_et_ ≤ 10. The results of tested samples of groups 1 and 2 (Table 5) show a similar course of changes as thermal resistance. In these two groups of samples, water-vapour resistance was more affected by the yarn fineness and fabric density. Samples 1a and 2b differ in the continuity of changes where the fabric mass and thickness prevailed, and provided greater resistance than the other samples. The results of water-vapour resistance in the other two groups of samples (3 and 4) with finer weft yarns show that the fabric mass and thickness had a greater influence in relation to the fineness and density of yarns in the weft system. The results of these samples show that greater fabric mass and thickness mostly provide greater water-vapour resistance.

Table 6 shows the relationships between the most relevant tested parameters, which prove the influence of individual parameters on final fabric properties, while Table 7 shows the significant values of those relationships between the mentioned parameters. The strength of the correlation in Table 6 is indicated by the colour intensity, so that the strongest correlations are shown in the most intense green, while the decrease in the correlation follows the decrease in the colour intensity. Thus, the influence of the proportion of individual yarns (defined by yarn fineness) on the fabric’s structural properties (mass and thickness) is clearly visible in Table 6. It is also important to point out that the influence of the quantitative proportion of aramid yarn is strongly related to thermal and thermo-physiological properties (TFQ_0_, R_et_), with great significance (*p* < 0.001). Furthermore, there is an extremely strong relationship between the fabric mass and the properties of resistance of radiant heat (TFQ_0_) and water-vapour resistance (R_et_), followed by extremely strong significance (*p* < 0.0001). It should also be noted that the analysed influences of structural parameters (m and t, and Tt_AR_) on the property of resistance of radiant heat (TFQ_0_) show a negative correlation, which means that their relationship is inversely proportional. Additionally, there is a significant relationship between the resistance of radiant heat (TFQ_0_) and water-vapour resistance (R_et_), where with a significance of *p* < 0.0001 there is an extremely strong inversely proportional relationship, i.e., negative correlation r = −0.84092.

By analysing the products available on the global market, which include single-layer woven fabrics (as one segment in the final protective clothing), it was determined that there is no multifunctional woven fabric that combines the key properties of non-flammability, comfort, and durability while maintaining a single-phase production process, and thus economical production. The above-mentioned available products face certain disadvantages, such as reduction of flammability, comfort, durability, heat protection, breathability, etc. The weight of such fabrics is between 200–250 g/m^2^, while the weight of the fabrics analysed in this research is from 300–420 g/m^2^. Despite the greater weight and thickness of the newly designed woven fabrics, they simultaneously achieve exceptional properties of thermal protection and comfort. The right selection of raw materials (inherently non-flammable aramid fibres and fire retardant modacrylic fibre with a half proportion of cotton), and a meticulously constructed complex structure, on one side (front) ensures thermal protection and strength, while on the other side (back) provides comfort while wearing. This achieves the property of multi-functionality with a single-phase production process and a reduction of production costs.

## 4. Conclusions

By designing a complex fabric construction, i.e., by applying a complex weave and yarns of different raw material compositions (AR and MC), a difference in structure and properties on the front and backside of the fabrics is achieved. Each raw material with its properties at the micro level contributes to the properties of the fabric at the macro level. Furthermore, the slightest change in the proportion of certain raw material in the totality of the fabric (changing the yarn fineness only in warp thread system) results in changes of properties at all levels. This gives a significant opportunity in designing woven fabrics with precisely defined target properties, depending on the end application. This complex woven construction achieves all set goals and meets requirements of thermal and thermo-physiological properties, without interventions in the form of layering, lamination or adding other materials for making composites. The combination of this complex weave, with the selection of AR yarns (which ensure good flame protection) and MC yarns (which have a stronger influence on the insulation properties and comfort), leads to the production of multi-functional woven fabric for protective clothing.

## Figures and Tables

**Table 1:** Declared parameters of the yarns.

Yarn Designation	Composition	Tt (Tex)	T (Twist/m)
			Single Yarn/Plied Yarn
AR	95% M-aramid Conex NEO; 5% P-aramid Twaron	20 × 2	740 Z/600 S
		17 × 2	840 Z/660 S
		14 × 2	940 Z/710 S
		12.5 × 2	1030 Z/760 S
MC	45% cotton fibre (combed); 55% modacrylic fibre Sevel FRSA/L	20 × 2	970 Z/670 S
		17 × 2	1000 Z/720 S
		14 × 2	1170 Z/800 S
		12.5 × 2	1200 Z/760 S

**Table 2:** Specification of the woven fabric samples.

Sample	Warp	Tt (Tex)	Weft 1	Tt (Tex)	Weft 2	Tt (Tex)
1a	AR	17 × 2	AR	20 × 2	MC	20 × 2
1b	AR	17 × 2	AR	20 × 2	MC	17 × 2
1c	AR	17 × 2	AR	20 × 2	MC	14 × 2
1d	AR	17 × 2	AR	20 × 2	MC	12.5 × 2
2a	AR	17 × 2	AR	17 × 2	MC	20 × 2
2b	AR	17 × 2	AR	17 × 2	MC	17 × 2
2c	AR	17 × 2	AR	17 × 2	MC	14 × 2
2d	AR	17 × 2	AR	17 × 2	MC	12.5 × 2
3a	AR	17 × 2	AR	14 × 2	MC	20 × 2
3b	AR	17 × 2	AR	14 × 2	MC	17 × 2
3c	AR	17 × 2	AR	14 × 2	MC	14 × 2
3d	AR	17 × 2	AR	14 × 2	MC	12.5 × 2
4a	AR	17 × 2	AR	12.5 × 2	MC	20 × 2
4b	AR	17 × 2	AR	12.5 × 2	MC	17 × 2
4c	AR	17 × 2	AR	12.5 × 2	MC	14 × 2
4d	AR	17 × 2	AR	12.5 × 2	MC	12.5 × 2

**Table 3:** Construction and structural parameters of the woven fabrics.

Designation	d_warp_ (Threads/cm)	d_weft_ (Threads/cm)	m (g/m^2^)	t (mm)
1a	34	64	417.72	1.372
1b	34	64	382.32	1.326
1c	34	66	367.11	1.322
1d	34	68	345.21	1.306
2a	34	58	356.42	1.418
2b	34	58	336.81	1.331
2c	34	60	316.83	1.293
2d	34	64	300.12	1.267
3a	34	68	359.29	1.272
3b	34	68	334.11	1.225
3c	34	69	300.78	1.194
3d	34	69	291.57	1.177
4a	34	64	322.93	1.304
4b	34	64	299.62	1.286
4c	34	64	281.88	1.259
4d	34	65	260.87	1.240

**Table 4:** Woven fabric resistance of radiant heat.

Designation	t_12_ (s)	t_24_ (s)	t_24_ − t_12_ (s)	Q_c_ (kw/m^2^)	TFQ_0_ (-)
1a	11.35	19.30	7.95	8.32	0.416
1b	10.70	18.25	7.55	8.38	0.420
1c	10.45	18.30	7.85	8.42	0.421
1d	10.15	18.00	7.85	8.42	0.421
2a	10.35	18.10	7.75	8.53	0.427
2b	10.25	17.70	7.45	8.88	0.444
2c	10.15	17.65	7.50	8.82	0.441
2d	9.95	17.30	7.35	9.00	0.450
3a	10.35	17.70	7.35	9.00	0.450
3b	9.80	16.95	7.15	9.25	0.462
3c	9.20	16.15	6.95	9.52	0.472
3d	9.45	16.45	7.00	9.45	0.473
4a	9.65	16.70	7.05	9.38	0.469
4b	9.00	16.00	7.00	9.45	0.472
4c	8.70	15.60	6.90	9.58	0.479
4d	8.15	14.90	6.75	9.80	0.490

**Table 5:** Thermal resistance (R_ct_) and water-vapour resistance (R_et_).

	1		2		3		4	
	R_ct_ (m^2^ K/W)	R_et_ (m^2^ Pa/W)	R_ct_ (m^2^ K/W)	R_et_ (m^2^ Pa/W)	R_ct_ (m^2^ K/W)	R_et_ (m^2^ Pa/W)	R_ct_ (m^2^ K/W)	R_et_ (m^2^ Pa/W)
a	0.0600	6.743	0.0578	5.541	0.0679	5.778	0.0549	5.796
b	0.0488	6.007	0.0589	5.907	0.0552	5.550	0.0632	5.505
c	0.0546	6.255	0.0566	5.566	0.0553	5.215	0.0631	5.095
d	0.0717	6.373	0.0651	5.828	0.0498	5.056	0.0577	5.208

**Table 6:** Correlation coefficient of relevant woven fabric parameters.

	Tt_AR_ (Tex)	Tt_MC_ (Tex)	d_weft_ (Threads/cm)	m (g/m^2^)	t (mm)	TFQ_0_ (-)	R_ct_ (m^2^ K/W)
Tt_MC_ (tex)	0	1					
d_weft_ (threads/cm)	−0.19334	−0.31745	1				
m (g/m^2^)	0.74953	0.60899	−0.13041	1			
t (mm)	0.59103	0.57859	−0.69997	0.68452	1		
TFQ_0_ (-)	−0.91757	−0.27287	0.28722	−0.84913	−0.74845	1	
R_ct_ (m^2^ K/W)	0.01385	−0.02961	0.04869	−0.02629	0.13988	−0.16584	1
R_et_ (m^2^ Pa/W)	0.80157	0.32778	−0.09980	0.83296	0.64134	−0.84092	0.25586

**Table 7:** *p*-values of correlated parameters.

	Tt_AR_ (Tex)	Tt_MC_ (Tex)	d_weft_ (Threads/cm)	m (g/m^2^)	t (mm)	TFQ_0_ (-)	R_ct_ (m^2^ K/W)
d_weft_ (threads/cm)	0.4731090	0.2308711					
m (g/m^2^)	0.0008295	0.0122841	0.6302461				
t (mm)	0.0159088	0.0188704	0.0025366	0.0034422			
TFQ_0_ (-)	0.0000006	0.3065377	0.2807575	0.0000317	0.0008522		
R_ct_ (m^2^ K/W)	0.9593906	0.9133260	0.8578728	0.9230067	0.6053683	0.5393198	
R_et_ (m^2^ Pa/W)	0.0001888	0.2152114	0.7130800	0.0000618	0.0074150	0.0000449	0.3388403

## Data Availability

Not applicable.

## Abbreviations

Designation	Description	Unit
A	area of the copper plate (0.002515 m^2^)	
AR	aramid fibre	-
C_p_	specific heat of copper (0.385 kJ/kg °C)	g
d_warp_	warp density	threads/cm
d_weft_	weft density	threads/cm
M	mass of the copper plate (0.036 kg)	g
m	fabric mass	g/m^2^
MC	modacrylic fibre	-
p-value	significance	
R	correlation coefficient	%
R_ct_	thermal resistance	m^2^ K/W
R_et_	water-vapour resistance	m^2^ Pa/W
T	twist	twist/m
t	thickness	mm
TFQ_0_	heat transmission factor	
Tt	fineness	tex
t_12_	time required for a temperature rise of 12 °C	s
t_24_	time required for a temperature rise of 24 °C	s
Q_c_	transmitted heat flux density, through the sample to the calorimeter	kw/m^2^
Q_o_	incident heat flux density level	
